# Metabolic and immune consequences of antibiotic related microbiome alterations during first-line tuberculosis treatment in Bamako, Mali

**DOI:** 10.3389/fimmu.2025.1561459

**Published:** 2025-05-14

**Authors:** Dramane Diallo, Shan Sun, Anou M. Somboro, Bocar Baya, Amadou Koné, Bassirou Diarra, Mohamed Nantoumé, Isaac Koloma, Mahamadou Diakite, Jane Holl, Almoustapha Issiaka Maiga, Moussa Seydi, Grant Theron, Lifang Hou, Anthony Fodor, Mamoudou Maiga

**Affiliations:** ^1^ University Clinical Research Center (UCRC), Bamako, Mali; ^2^ Department of Bioinformatics and Genomics, University of North Carolina at Charlotte, Charlotte, NC, United States; ^3^ Antimicrobial Research Unit, College of Health Sciences, University of KwaZulu-Natal, Durban, South Africa; ^4^ University of Sciences, Techniques, and Technologies of Bamako (USTTB), Bamako, Mali; ^5^ Department of Neurology and Center for Healthcare Delivery Science and Innovation, University of Chicago, Chicago, IL, United States; ^6^ Service des Maladies Infectieuses et Tropicales, Fann University Hospital Center, Dakar, Senegal; ^7^ Centre of Excellence for Biomedical Tuberculosis Research, South African Medical Research Council Centre for Tuberculosis, Stellenbosch University, Cape Town, South Africa; ^8^ Feinberg School of Medicine, Northwestern University, Chicago, IL, United States

**Keywords:** tuberculosis, gut microbiome alterations, metabolic and immune response, tuberculosis treatment, dysbiosis, Mali

## Abstract

**Background:**

Individuals with a history of tuberculosis (TB) treatment are at a higher risk of experiencing a recurrent episode of the disease. Previous cross-sectional studies identified a connection between dysbiosis (alterations) in the gut microbiota composition and the administration of first-line TB antibiotics. However, these studies have not successfully elucidated this dysbiosis’s resulting metabolic and immune consequences.

**Methods:**

In a longitudinal assessment, we studied the antituberculosis drug-related changes in the gut microbiota’s composition and the resulting functional consequences. Sputum for TB culture, peripheral blood for metabolomics and cytokines analysis, and stool for shotgun metagenomics were collected from TB participants at Month-0, Month-2, Month-6 of treatment, and 9 Months after treatment (Month-15). Healthy controls were sampled at Month-0 and Month-6.

**Findings:**

We found notable differences in gut microbiota between individuals with TB and healthy controls. While gut microbiota tended to resemble healthy controls at the end of TB treatment, significant differences for many taxa persisted up to Month-15. Concurrently, disturbances in plasma metabolites, including tryptophan, tricarboxylic acids, and cytokine levels were observed. Certain fatty acids associated with inflammation pathways negatively correlated with the abundance of several taxa.

**Conclusion:**

We observed alterations in the gut microbiota composition and function during treatment and at Month-15. Numerous changes in bacterial taxa abundances and inflammation-linked metabolites did not reverse at Month-15. This study suggests potential influences of anti-TB drugs and the gut microbiome on the disease outcome, response to treatment, and resistance to future TB infections.

## Background

Tuberculosis (TB) remains ranked as one of the leading causes of death from a single pathogen, *Mycobacterium tuberculosis (Mtb)*, with 1.13 million deaths among HIV-negative patients and 167,000 deaths among HIV-positive patients reported only in 2022 (1). One-fourth of the world’s population has been infected with *Mtb*, and more than 10 million people develop the disease yearly (1). Multiple underlying environmental conditions and immune and host genetic predisposing factors have been associated with TB infection (2, 3). Furthermore, treatment duration, adverse events with antibiotics, and drug resistance are important factors for disease outcomes. Standard first-line TB treatment requires six months of combination treatment with isoniazid, rifampicin, ethambutol, and pyrazinamide (4). However, treated and cured individuals are at least 8 times more likely to experience a new episode of TB disease than the general population (5–7).

Disruption of gut microbiota, which includes bacteria, archaea, and fungi, is a key factor potentially contributing to TB recurrence (8–10). Previous studies, many of which are cross-sectional, have found that TB and its treatment cause long-lasting dysbiosis up to two years after treatment. However, the metabolic consequences remain unexplored (5, 11). In addition, the microbiome-linked functional changes have not been studied longitudinally during TB treatment.

The Gut microbiota produces metabolites, such as short-chain fatty acids (SCFAs), that contribute to the host’s overall metabolic function, including defense against pathogens and drug metabolism (12). Anaerobic commensals produce enzymes that degrade dietary fibers into SCFAs (such as acetate, propionate, and butyrate), which regulate host immune-inflammatory response.

We conducted a longitudinal clinical study to investigate changes in gut microbiota profiles in TB patients before, during, and after treatment and in healthy controls, using shotgun metagenomics, metabolomics, and human Th1/Th2/Th17 cytometric bead array (CBA) for cytokines measurement. This study is one of the first of its kind to use shotgun metagenomics to understand the effect of anti-TB treatment on gut microbiota over time. This study provides new knowledge about potential strategies to improve TB treatment efficacy using host microbiota-directed therapies (10).

## Methods

### Study design and setting

We conducted a longitudinal study from February 2016 to August 2020, enrolling newly diagnosed TB cases based on sputum smear-positive with AFB (acid fast bacilli) from TB referral health centers in Bamako, the capital city of Mali, West Africa, and then later confirmed by culture at the University Clinical Research Center, University of Sciences Techniques and Technologies of Bamako (USTTB), Mali. In this longitudinal cohort study, a total of 155 TB patients were enrolled. However, were included in this final analysis only 30 TB participants, who were infected with *Mtb* complex strains confirmed by phenotypic (*Mtb* culture) and genotypic (Spoligotyping) identification methods.

### Study subjects and samples processing

Participants in this study included a group of TB patients and healthy individuals. *Mtb*-infected participants had four study visits for clinical investigation and sample collection as follows: before TB treatment initiation (TB_M0), two months (TB_M2), and six months (TB_M6) during anti-tuberculosis therapy, and then nine months after treatment completion (TB_M15). Healthy controls were sampled at Month-0 and Month-6 during the recruitment periods of TB patients. Samples collected include sputum, plasma, and stool from each participant. All the participants were 18 years and above who were newly diagnosed microscopically with pulmonary tuberculosis (TB group and later confirmed with sputum culture and molecular identification) and healthy control volunteers (Control group), with no TB disease or no latent TB infection as confirmed by the QuantiFERON-TB Gold assay (QFT-Plus; Qiagen, Hilden, Germany). All the participants were confirmed HIV seronegative and without any prior anti-tuberculosis therapy or antibiotics in the past 4 weeks prior to enrollment. Of all the TB patients included, 23 (76.6%) completed the study in Month-15, while 26 healthy controls provided the requested samples for the study. All the participants (TB and Healthy cases) were from the same geographical region (Bamako, the capital city of Mali in West Africa). A written and signed informed consent form was obtained from each participant before being enrolled. The confirmed cases of the newly diagnosed TB patients were followed before and after starting a first-line anti-tuberculosis treatment regimen comprising two months of isoniazid (H), rifampin (R), pyrazinamide (Z), ethambutol (E), followed by four months of rifampin (R), and isoniazid (H) (2HRZE/4RH).

### Ethics approval and consent to participate

The study protocol was approved by the Institutional Review Board (IRB) of Northwestern University (approval number: STU00094500) of Chicago (USA) and the Ethics committee of the University of Sciences Techniques and Technologies of Bamako (USTTB), Mali (approval number: 2014/04 CE/FMPOS). A consent form was signed by each participant before inclusion in the study.

### Mycobacterial identifications

Early morning sputum specimens collected from presumptive TB patients were tested for TB using the standard N-acetyl-L-cysteine/4% sodium hydroxide solution for sputum digestion and decontamination; thereafter, the sample was concentrated by high-speed centrifugation. The pellets were used to inoculate liquid medium (Mycobacterium Growth Incubator Tube (MGIT™) [BD, Sparks, MD, USA], and solid medium (Middlebrook 7H11 agar and selective 7H11 agar) to isolate pure mycobacteria colonies, as previously described (13, 14).

Confirmed *Mtb* isolates underwent Spoligotyping to determine the mycobacterial strains. Therefore, DNA from pure isolates of *Mtb* were extracted, and Spoligotying was performed according to the manufacturer’s instructions using commercial kits (Isogen Bioscience BV, Maarssen, The Netherlands). A detailed description of the Spoligotyping methodology can be found in the supplementary data.

### Cytokine measurements

Cytokine levels were monitored longitudinally to assess the immune response from the pre- to post-treatment stages. Per the manufacturer’s instructions, we used the Human Th1/Th2/Th17 kit (BD Biosciences, San Diego, USA) to perform the cytokine measurement assays. The BD CBA assessed Interleukin-2 (IL2), IL4, IL6, IL10, TNF, IFN-γ, and IL17A levels in plasma samples. Samples were thawed at 4-8°C before the assay, then prepared and measured following instructions using an LSR II flow cytometer at the University Clinical Research Center, Mali (UCRC). Analysis used FCAP Array TM software v3.0.1 and the detection limits were IL-2 (2.6 pg/mL), IL-4 (4.9 pg/mL), IL-6 (2.4 pg/mL), IL-10 (4.5 pg/mL), TNF (3.8 pg/mL), IFN-γ (3.7 pg/mL), and IL-17A (18.9 pg/mL).

### DNA extraction from stool samples

DNA was extracted from stool samples using the QIAmp DNA Stool Mini Kit (Qiagen, Hilden, Germany). Frozen stool samples were placed and thawed on ice before the extraction starts. The stool samples were first weighed (180–220 mg of thawed stool), thereafter, extraction was performed according to the manufacturer’s instructions (QIAmp DNA Stool Mini Kit (Qiagen, Hilden, Germany)). The extracted DNA was then measured using the Nanodrop One^c^ (Thermofisher Scientific, Verona Rd, Madison, USA) to assess the DNA concentration and purity before sequencing.

### Shotgun metagenomics of the gut microbiome and bioinformatics analysis

DNA from stool samples was sequenced at the University of Illinois at Chicago (UIC) Research Genome Core Laboratory employing Illumina HiSeq 4000 using 150 bp paired-end reads. The taxonomic composition was profiled using Kraken2, and the functional pathways (stratified and unstratified) were characterized using HUMAnN2 per developers’ instructions. The sequencing reads ranged from 496,978 to 31,329,790 across 122 samples, with an average 20,311,846 reads per sample. The human reads were detected by alignment to human genomes and removed with KneadData from bioBakery. The proportion of reads aligned to human genomes ranged from 0.001% to 6.33%, with an average of 0.27%. On average, 6,681,981 reads/sample were classified to bacteria, 114,108 reads/sample were classified to archaea, 14,876 reads/sample were classified to fungi, 1,480 reads/sample were classified to non-fungal Eukaryota and 410,915 reads/sample were classified to virus.

The statistical analysis of taxonomic composition and pathway abundances were performed with R. The Principal Coordinate Analysis (PCoA) was calculated based on the Bray-Curtis dissimilarity using ‘capscale’ function in the R package ‘vegan’. Alpha diversity was measured using Shannon index while beta dispersion between groups was analyzed using TukeyHSD. The differential taxa and pathways between healthy controls and TB patients at each time point were analyzed with non-parametric Wilcoxon test and linear regression models adjusted for age. The change of taxa and pathways with time in TB patients were analyzed with linear mixed-effects models with continuous time as main effect and subject ID as random effects. P values were adjusted using the Benjamini-Hochberg method for multiple hypotheses testing. False Discovery Rate (FDR) <0.1 was considered as statistically significant.

### Metabolomics analysis of plasma samples

Metabolomic analysis was performed on the plasma samples from TB patients only at three time points (TB_M0, TB_M6, and TB_M15) at Metabolon (Morrisville, NC, USA). Samples were prepared using the automated MicroLab STAR^®^ system from Hamilton Company. The extracts were analyzed with reverse phase (RP)/UPLC-MS/MS in positive ion mode electrospray ionization (ESI), RP/UPLC-MS/MS with negative ion mode ESI and HILIC/UPLC-MS/MS with negative ion mode ESI. All methods utilized a Waters ACQUITY ultra-performance liquid chromatography (UPLC) and a Thermo Scientific Q-Exactive high resolution/accurate mass spectrometer interfaced with a heated electrospray ionization (HESI-II) source and Orbitrap mass analyzer operated at 35,000 mass resolution. Raw data was extracted, peak-identified and QC processed using Metabolon’s equipment and software with peaks quantified by area-under-the-curve. The metabolomics data were mapped to Metabolon’s biochemical pathways to analyze the changes of functional pathways. Detailed methods can be found in supplementary information. Principal components analysis (PCA) was used to visualize the differences in metabolome profiles between time-points. Hierarchical Clustering Analysis (HCA) was used to cluster samples based on the Euclidean distance. ANOVA with repeated measures analyzed differential metabolites across groups with contrasts revealing significant differences between each pair. The association between taxonomic abundance and metabolites were analyzed with Spearman’s correlation. P-values were adjusted using the Benjamini-Hochberg method to correct for multiple testing. GraphPad prism version 8.0.1 was used for patients’ social characteristics analyses and cytokines measurement analysis.

## Results

### Participants’ socio-demographic and clinical characteristics

The longitudinal cohort study involved thirty (30) confirmed TB individuals and twenty-six (26) healthy controls (TB and HIV negative). The TB-infected participants were followed up for over 15 months, including during six months of the treatment and then nine months after the treatment completion. The age, sex, and smoking status of study participants are shown in **Table 1**. Age was significantly different between healthy controls and TB patients, while sex and smoking status were not significantly different (**Table 1**). A total of 19/30 (63.33%) of the TB patients were infected with the modern Euro-American lineage 4, which showed a high amount of persistence of sputum smear positivity at month-2 of TB treatment 10/13 (76.92%). The other circulating lineages in Mali, lineage 1, lineage 2, and lineage 3, were more responsive to the anti-TB drugs, 8/11 (72.72%) at this time point.

**Table 1:** Characteristics of study participants.

Participants Characteristics	Case	Control	P value^1^
Number	30	26	
Age (y), mean (SD)	32.5 (11.8)	25.8 (5.7)	0.012
Female, %	16.7	34.6	0.14
Non-smoking %	66.7	76.9	0.55

^1^For age, p-value derived from Wilcoxon test. For sex and smoking, p-value derived from Fisher’s exact test.

### Longitudinal metagenomics analysis of microbial diversity in the gut of TB patients during drug treatment

**Figure 1:**
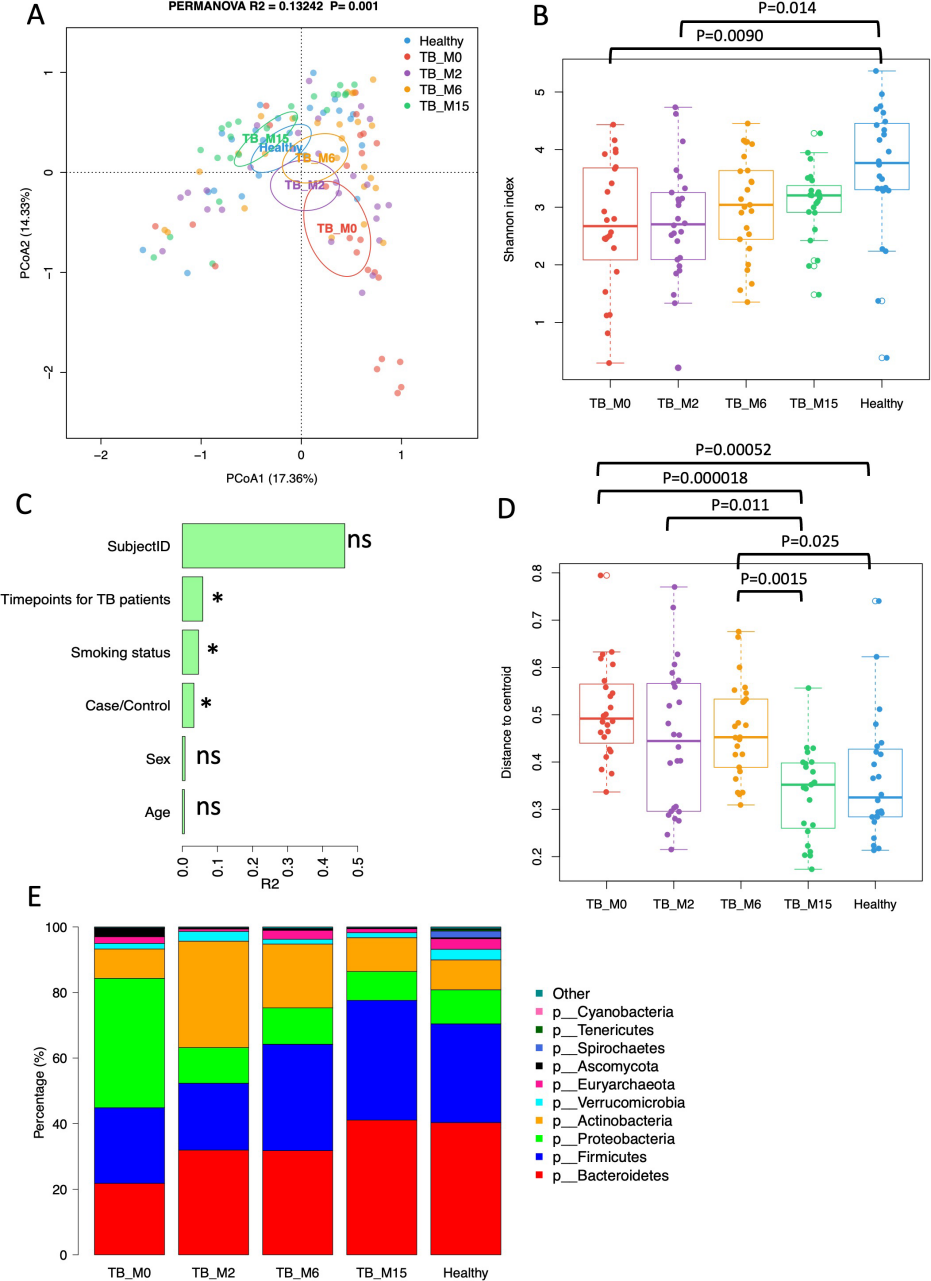
Gut microbiota diversity and taxonomic composition of TB patients and healthy controls. **(A)** Principal Coordinates Analysis (PCoA) of the microbial communities at the genus level. Cases are colored by groups, and time points and ellipses indicate 95% confidence limits. **(B)** Shannon diversity of TB patients and healthy controls. Each point refers to an individual sample. **(C)** PERMANOVA R2 of the association between microbial taxonomic composition and metadata. **(D)** Beta-diversity (distance to centroid) of TB patients and healthy controls. Each point refers to an individual sample. **(E)** Bar plot of average phylum composition of TB patients at each time-point and healthy individuals. * statistically significant.

PCoA at the genus level showed the separation of gut microbial communities by study groups (**Figure 1A**). For longitudinal analysis, *Mtb*-infected individuals were shown by time-points: TB_M0, TB_M2, TB_M6, and TB_M15, as described above. The gut microbiota of TB_M0 and TB_M2 were distinct from healthy controls. While TB_M6 and TB_M15 microbiota became more similar to healthy controls but are still significantly different (PERMANOVA test: TB_M6 vs Healthy: R²=0.065, P=0.002; TB_M15 vs Healthy: R²=0.087, P=0.001). The differences between TB patients and healthy controls remained significant after adjusting for age in the PERMANOVA model (P=0.001). Shannon diversity increased in patients’ gut microbiota over time, with TB_M0 and TB_M2 significantly different from healthy controls and TB_M6 and TB_M15 insignificant (TukeyHSD, ANOVA) (**Figure 1B**). We tested the association of microbial community and metadata, including Case/Control, time points for patients, age, sex, smoking status, and subject ID using a univariate PERMANOVA test. We found that Case/Control, time points for patients, and smoking status were significantly associated with the microbial community in this cohort (P<0.05), while age, sex, and subject ID were not significantly associated (**Figure 1C**). Among the significant associated metadata, time points in patients have the largest effect size (R2). The beta-diversity of TB_M0 and TB_M6 were significantly higher than that of healthy controls and TB_M15, while the beta-diversity of TB_M15 is similar to that of healthy controls (**Figure 1D**).

In addition to PERMANOVA tests, which examine beta diversity metrics on the entire microbial community, we analyzed the differences in relative abundance for taxonomic composition between patients and controls using the Wilcoxon test and linear regression models adjusted for age. We also analyzed the changes in treatment time with mixed effects linear models with time as the main effect and subject ID as the random effect in patients. At the phylum level, Firmicutes increased significantly with time after treatment, while Proteobacteria decreased significantly (**Figure 1E**), (linear mixed effect models, FDR<0.1). In patients, 411 taxa across taxonomic levels from phylum to species increased with time, and nine decreased with time (linear mixed effect models, FDR<0.1, (**Supplementary Table S1**)). At M15, Lactobacillus and Lactobacillaceae were still significantly lower than that of healthy controls (Wilcoxon test, FDR<0.1, (**Supplementary Table S1**)). In patients, 815 taxa were significantly lower than healthy controls at all four-time points, and two taxa were significantly higher than healthy controls (**Supplementary Table S1**). After adjusting for age, 1234 taxa were significantly less abundant at TB_0 than healthy controls, among which 767 were still less abundant at TB_2. However, none of these were still significant at TB_6 and TB_15 after age adjustment (**Supplementary Table S2**). At TB_0, 32 taxa were significantly more abundant than healthy controls, but none of these taxa were still significant after treatment at TB_2 (**Supplementary Table S2**).

**Figure 2:**
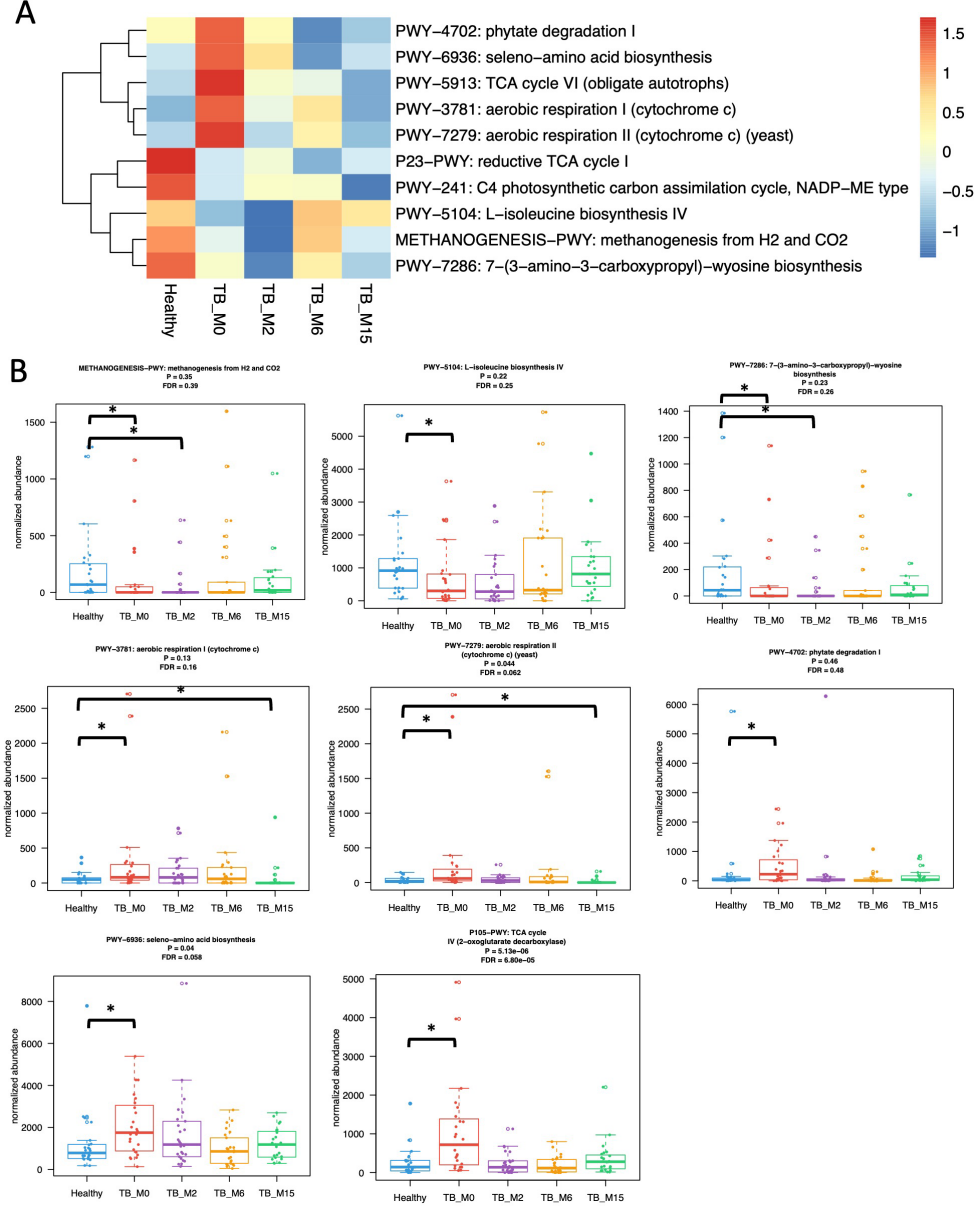
Differences in gut microbial functional pathways between TB patients and healthy subjects. **(A)** Heatmap shows select pathways significantly different between healthy controls and TB patients before treatment with the Wilcoxon test adjusted for multiple hypotheses testing. Pathways present in <25% of samples are excluded from the analyses. Keys indicate z-scores of relative abundances. **(B)** Box plots showed the changes in eight significant functional pathways. * statistically significant.

We analyzed the differences in functional pathway abundance between TB patients and healthy controls with Wilcoxon test and linear regression models adjusted for age and the changes in functional pathways with treatment time with mixed effects linear models with time as the main effect and subject ID as the random effect. (**Figure 2**) In TB patients, 75 pathways increased with time, and 11 decreased with time (linear mixed effect models, FDR<0.1, **Supplementary Table S3**). Compared with healthy controls, 11 pathways were less abundant at TB_M0, and 212 pathways were more abundant. Among these pathways, four were still less abundant, and 15 were still more abundant at TB_M2. None of these pathways were significantly different between TB_M6 and controls, while three became significantly more abundant at TB_M15 again. After adjusting for covariates, 207 pathways were significantly more abundant at TB_M0 than healthy controls, and two were significantly less abundant, among which none were significant at TB_M2 (**Supplementary Table S4**).

### Metabolomics analysis

Three groups were compared to evaluate changes in blood metabolites: TB_M0, TB_M6, and TB_M15. By using ANOVA with repeated measures, 470 metabolites were found to be statistically significant across the three groups (FDR<0.1). Next, we compared each pair of groups with ANOVA contrast and found that 361 metabolites were significantly different between TB_M0 and TB_M6, 103 metabolites were significantly different between TB_M6 and TB_M15 and 428 metabolites were significantly different between TB_M0 and TB_M15.

**Figure 3:**
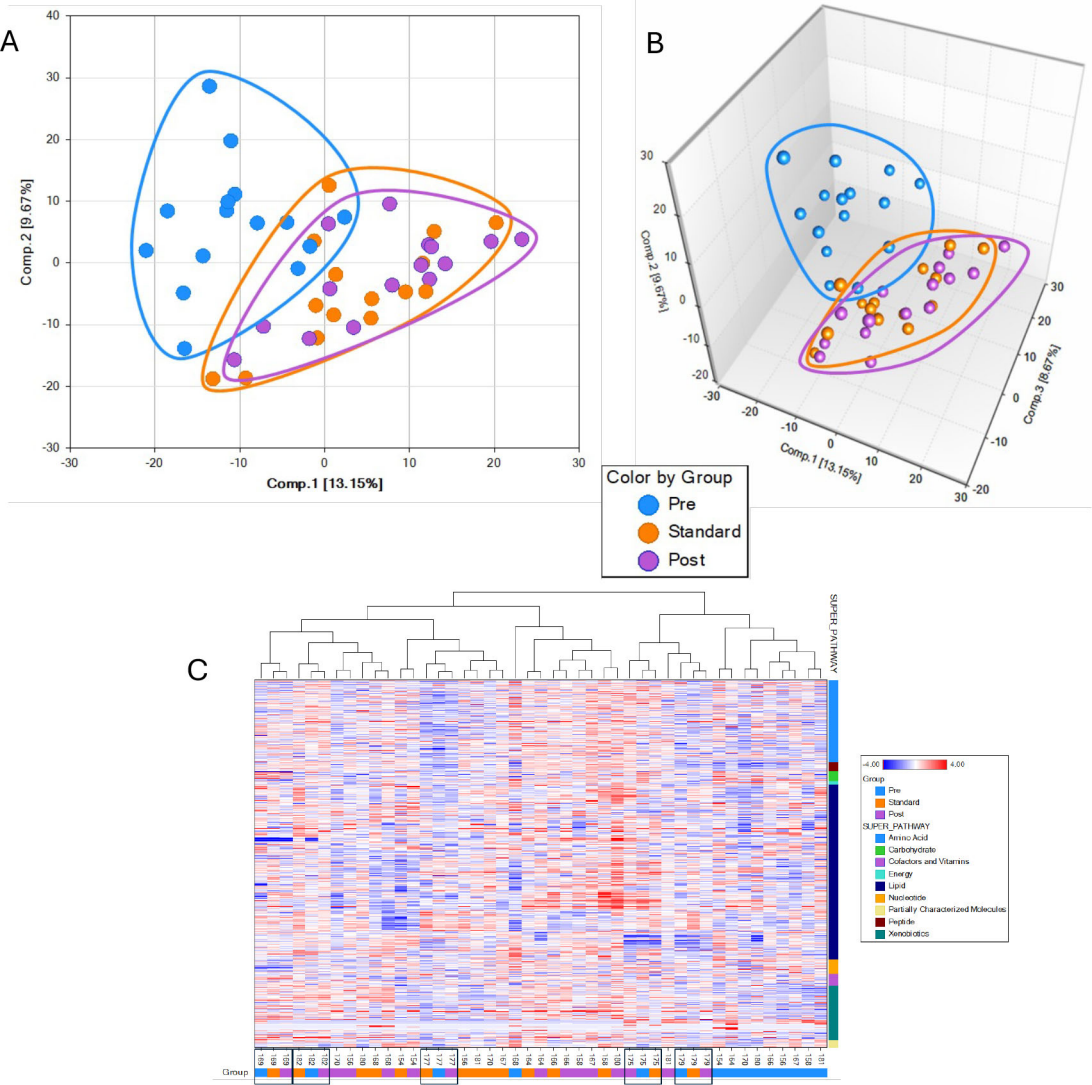
Principal component analysis showing separation in metabolomics profile of the different groups. Data are displayed in two dimensions: **(A)**, three dimensions **(B, C)** Hierarchical clustering analysis of metabolite pathways from plasma.

Principal Components Analysis (PCA) plots showed that TB_M0 is distinct and separated from TB_M6 and TB_M15, indicating that the treatment effects on metabolites were maintained at least nine months after treatment completion (**Figures 3A, B**).

The overlap between TB_M6 and TB_M15 suggests their metabolic profiles are similar and different from the pre-treatment/infection stage. Using the Hierarchical Clustering Analysis (HCA), we found five groupings of 15 subjects’ samples of metabolic profiles (**Figure 3C**). A cluster of nine pre-treatment samples also suggests good similarities among many pre-treatment samples. The lack of such high clustering at the end of the standard treatment and nine months after treatment suggested that the variance from these two-time points is smaller than the variance across the study subjects.

**Figure 4:**
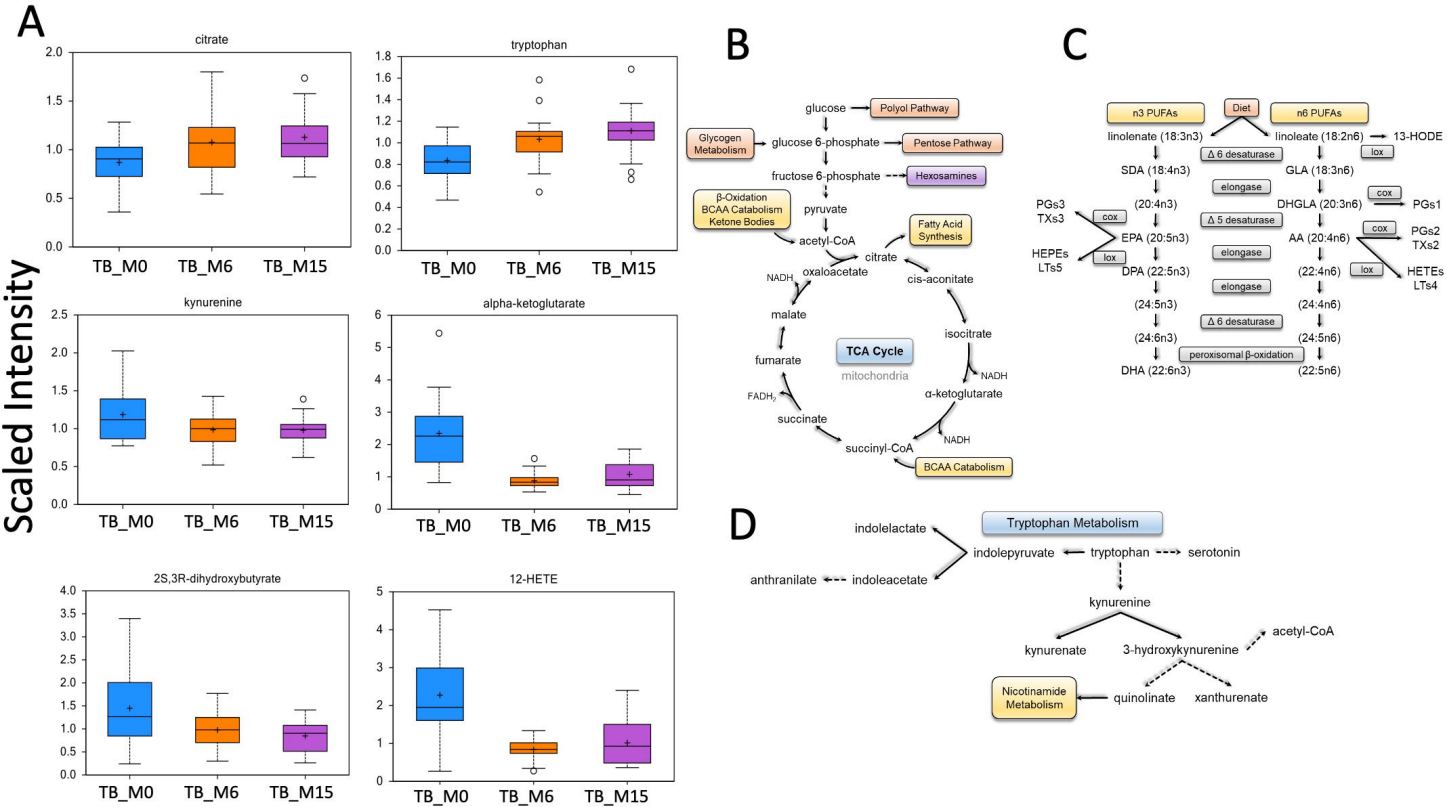
Main biochemical pathway changes during treatment of TB disease. Metabolomics data showed changes in the scaled intensity of biochemicals.

Our data showed major changes between TB time points in four main pathways, as reported in (**Figure 4**), such as tryptophan metabolism, fatty acid metabolism, and energy pathways.

### Correlation between genus and pathways abundance and produced microbial metabolites

**Figure 5:**
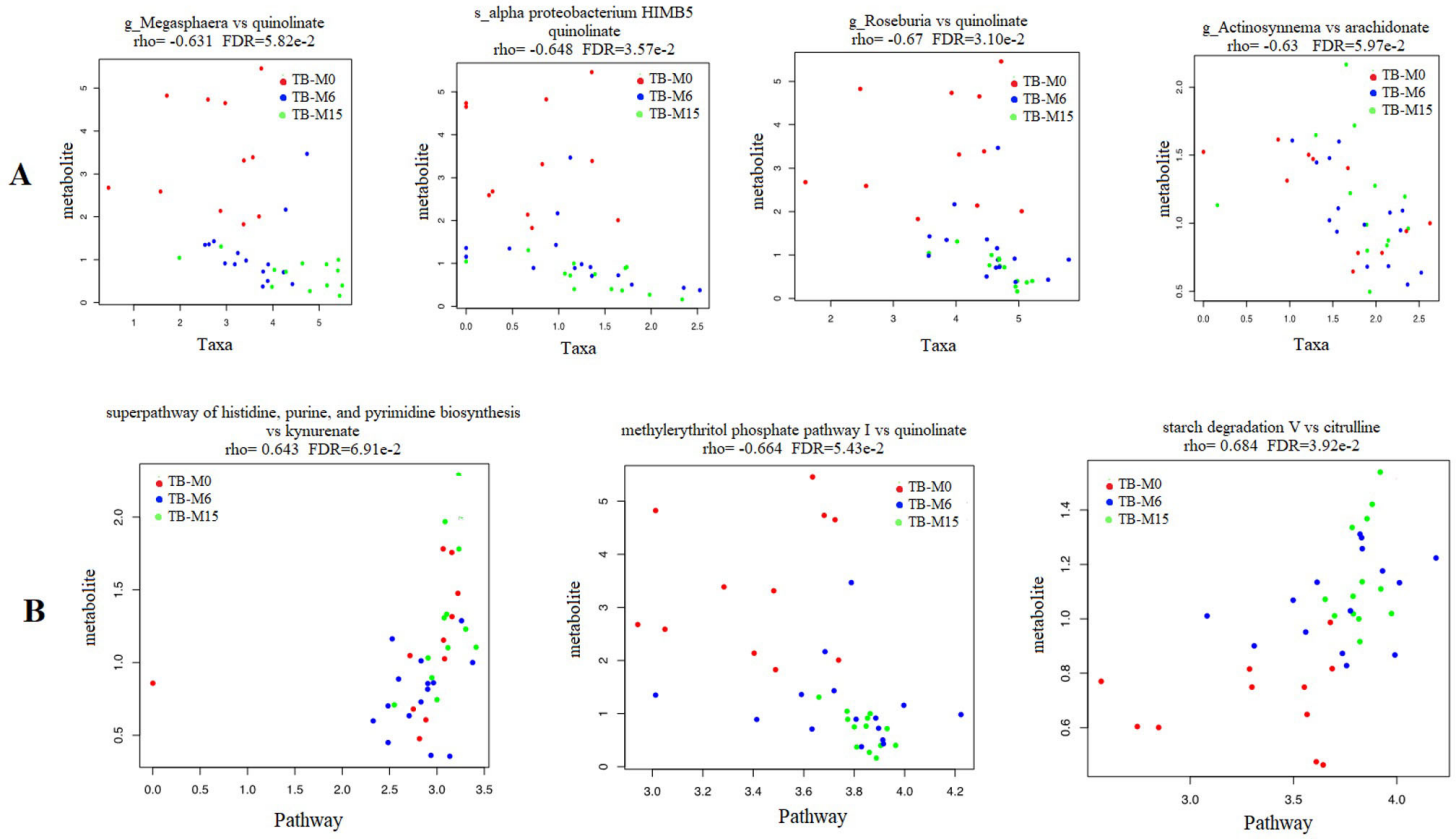
Correlation between microbial-produced metabolites and both pathways and taxa abundance. **(A)** Quinolinate was negatively correlated with the abundance of genera Megasphaera, Roseburia and the species Alpha proteobacterium HIMB5 from the gut microbiota. Arachidonate was negatively correlated with the abundance of genus Actinosynnema. **(B)** Citrulline and kynurenate were found to be positively correlated with starch degradation V and histidine, purine and pyrimidine biosynthesis pathways respectively, while the quinolinate was negatively correlated with methylerythritol phosphate pathway I.

We found significant correlations between genus/pathway and metabolite abundance in the gut during the six-month TB antibiotic regimen. We compared the metabolites’ production with the taxa’s relative abundance TB_M6 and TB_M15 (**Figure 5A**). The whole correlated taxa from gut microbiota to peripheral metabolites are listed in supplemental data 1. The taxa correlated with metabolite production belong to the bacteria phyla of Proteobacteria, Actinobacteria, and Firmicutes, and Euryarchaeota from the archaea domain. The fatty acids such as quinoline and arachidonate levels that are important for the inflammatory balance/pathway decreased with the increase of these taxa. In addition, we found correlations between the pathways’ alterations and produced metabolites (**Figure 5B**). A negative correlation was found between quinolinate and both Guanosine and methylerythritol phosphate pathway I. The ornithine was also found to negatively correlate with the alteration of glycolysis, tricarboxylic acid cycle, and glyoxylate bypass. In addition, citrulline and kynurenate were found to be positively correlated with the alteration of histidine, purine, and pyrimidine biosynthesis (kynurenate), aromatic amino acid biosynthesis, and starch degradation V pathway (citrulline) in the samples of TB patients.

### Cytokines analysis with CBA

**Figure 6:**
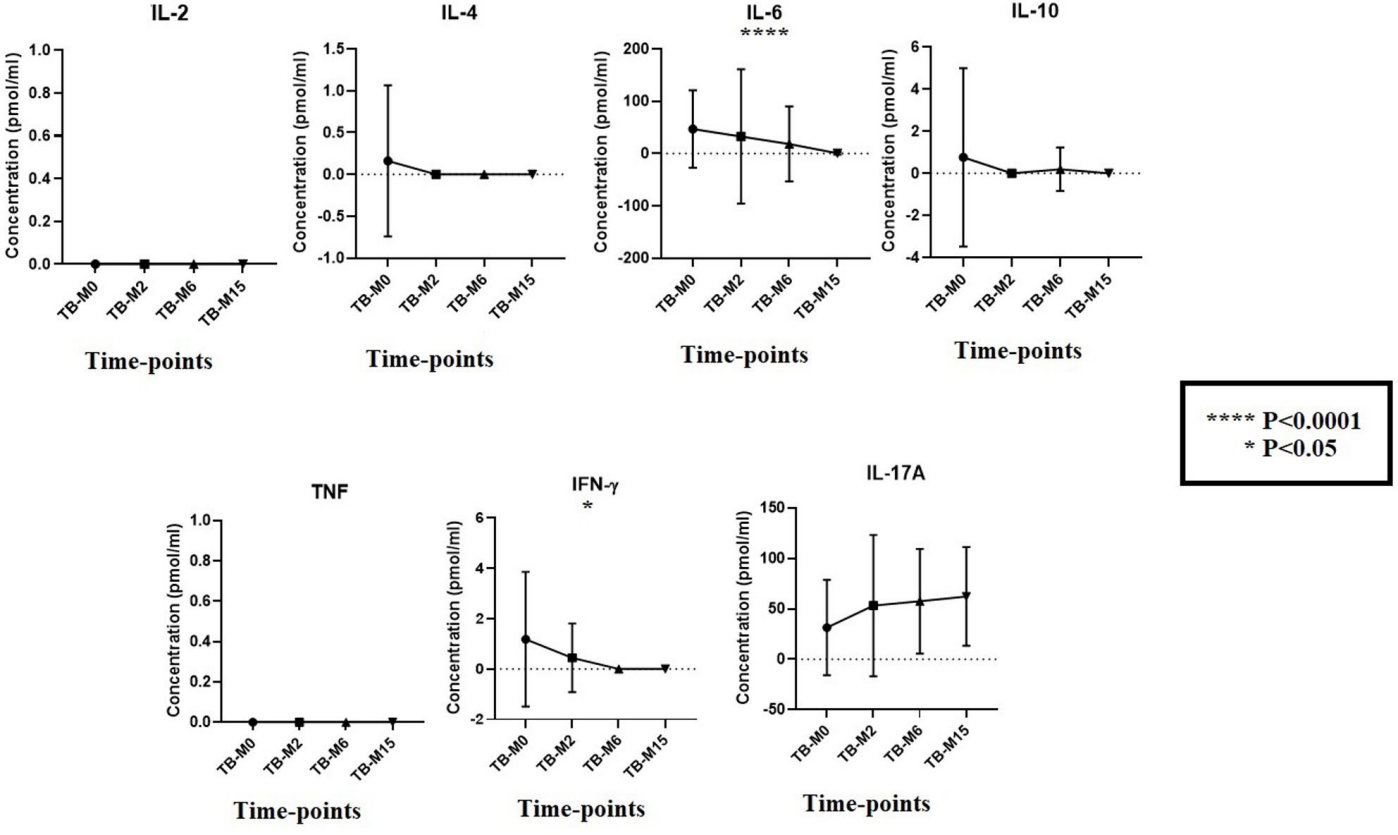
Cytokines production at study time points for TB groups. To measure the kinetics of cytokines during the disease in patients, we measured their concentrations longitudinally in plasma (TB_M0, TB_M2, TB_M6, and TB_M15). We observed a degradation of cytokines when comparing the values to other studies, which does not change the trend of the kinetics. IL-6 and IFN-γ were statistically significant (P<0.0001 and P<0.05, respectively). For IL-4 and IL-10, a reduction in their levels was observed after two months of treatment.

The immunological profile of participants was analyzed using the cytokines levels that are relevant from the literature for both TB disease and the microbiome (**Figure 6**). Despite the concentrations below the analysis’s detection limit, we compare the trend of cytokines from patients during the study time points without healthy individuals. We found that the mean cytokines levels were high for the major inflammatory players that are important for TB disease, such as IL-4, IL-6, IL-10, and IFN-γ TB-M0, but the levels continued to decrease slowly during and after treatment completion. In contrast, IL-17A, known to have a strong link with the gut, was highly expressed during the treatment period, and the trend was maintained a long time after completion (TB-M15).

## Discussion

This longitudinal cohort study looked at the gut microbiota profiles dynamics of TB patients before, during, and after treatment and thereafter compared to healthy individuals, this includes the microbial and metabolite changes within TB patients at different treatment stages and between TB and healthy participants. The study revealed that the gut microbiome of TB patients before and after treatment was distinct from that of healthy individuals, and the altered microbial community in the gut environment persisted for at least nine months after treatment completion. Gut microbiota is involved in the biological homeostasis of the host by its implication in the production of molecules that interact with the host cells. The dysregulation likely due to the *Mtb* infection tends to become normal after treatment (5, 15, 16). However, individuals with successful TB treatment outcomes remain at risk of developing the disease again (recurrent TB), either from the initial *Mtb* strain (relapse) or from a new strain (reinfection). Our study showed that the use of anti-mycobacterial drugs is also associated with human gut microbes disruption leading to gut microbiome dysbiosis. Therefore, the gut microbiome dysbiosis induced by anti-tuberculosis treatment may contribute to the risk of recurrent TB. Previous studies supported this hypothesis regarding treated TB-infected individuals having higher chances of developing a new TB episode (7, 17, 18), and this could be explained by the long-lasting damage from TB drugs on the gut microbiota and the resulting impact of its homeostatic role in inflammation and other metabolic functions that are essential for resistance against *Mtb*.

Recent studies showed significant relationships between changes in the gut microbiota and many human disease outcomes, including tuberculosis (19–21). The gender of men represents the majority of TB patients in our study, and that was also reported in several other studies (11, 22, 23). The mean age for TB patients was 32.5, and the smoking status represented 33%. Studies related to TB infection revealed its impact on young men and its association with cigarette use (14, 24, 25).

Because of the high percentage of bacteria in gut microbiota composition, other microbes, such as archaea, viruses, and fungi, are also being impacted during TB treatment but are generally less investigated compared to bacteria. Our study findings showed significant differences in the Euryarchaeota phylum from the archaea domain. One of the two major known archaeal phyla, Euryarchaeota, decreased from the gut microbiota of TB_M0 and at TB_M2 (with isoniazid and rifampicin regimen), then the level increased at TB_M6 before being finally decreased at TB_M15.

As reported by Negi S et al. in the mice model, the use of broad-spectrum TB antibiotics, such as rifampicin active on Gram-positive bacteria, could contribute to changes in the gut microbiota diversity and composition (26). In our study, the persistence of the alteration lasted nine months after treatment completion, and the data showed a negative correlation between the genus Actinosynnema, Megasphaera, and Roseburia relative abundances and the quinolinate metabolite. Quinolinate (quinolinic acid) is known as a marker for kynurenine in the tryptophan pathway (27). Similarly, Shibata et al. also reported disturbing effects after administration of antituberculosis drugs (mainly pyrazinamide and isoniazid) on the metabolic pathways of quinolinic acid (28). Furthermore, the kynurenine/tryptophan ratio is reported to be a great biomarker for pulmonary tuberculosis (29). Antibiotics during TB treatment potentially caused dysbiosis in the mentioned genus, leading to alterations in the plasma tryptophan pathway. Furthermore, our study found that arachidonic acid, a polyunsaturated fatty acid, negatively correlates with the abundance of Actinosynnema. This acid, used by *Mtb* via biosynthesis in infected macrophages, impairs their inflammatory and antimicrobial activities; its persistence nine months after TB treatment may explain the higher vulnerability of cured TB patients to new TB episodes, up to eight times more than the general population (30).

We further measured the level of seven cytokines from the plasma of TB patients, performed a comparative analysis between time points, and observed that IL-6 and IFN-γ were the most significantly higher levels of cytokines, which play an important role in the acute phase response against *Mtb*, while later they may potentiate tissue damage and induce other pathological pathways (31, 32). During TB treatment, we observed a decrease in their levels before the total bacterial clearance predicting positive TB treatment outcomes. Moreover, the gut microbiome also influences cytokines production, both directly via metabolites and indirectly by modulating host immune cells, which has an impact on immune homeostasis and disease susceptibility (33).

This study has some limitations: The sample size could be bigger to establish a stronger statement regarding this study’s conclusion, although the size used here is in line with other similar conducted studies (11, 15). Future studies must include various populations with a bigger sample size to generalize the findings. Participants were not matched by age and gender. However, when we analyzed the differences in these two parameters between case and control, we found that age was significantly different. We, therefore, adjusted for age in some of our statistical models. In addition, the healthy group was sampled at a different time than the TB patients and some of the differences between the cohorts may be explained by batch effects associated with these differences.

Another limitation of our study is that while we have control data from the microbiome, we did not collect metabolome or cytokines information for healthy controls. Finally, the effects of the TB drugs were measured collectively for multiple TB drugs and not individually, as it is not ethical to treat patients with a single drug. However, our studies in animal models in the past have found that rifampin, a large spectrum antibiotic, was responsible for the majority of dysbiosis seen with this drug regimen (5). Despite these limitations, and based on recent reports, this study is the most comprehensive analysis of the consequences of TB drug-related dysbiosis in participants during and after treatment and will advance the field of TB microbiome and our understanding of involved mechanisms.

In conclusion, the gut microbiota dysbiosis caused by antituberculosis drugs persists up to nine months after treatment completion. It shows putative links between microbiota-related metabolites and their pathways, which may contribute to weakening the inflammatory balance in TB-treated participants, and this could potentially make them more vulnerable to another TB episode. It is essential to characterize the dynamics of the gut microbiome and its metabolites during TB treatment first to improve treatment efficacy using host microbiota-directed therapies and, second, to prevent recurrent tuberculosis.

## Data availability statement

Sequences from this study are available on NCBI under the accession number PRJNA1002577 (https://www.ncbi.nlm.nih.gov/bioproject/PRJNA1002577).
